# Incubation of social deficit during morphine abstinence in male mice using a novel unbiased and automatized method

**DOI:** 10.3389/fnbeh.2025.1697469

**Published:** 2025-10-29

**Authors:** Victor P. Mathis, Gabriele Giua, Nicolas Torquet, Christophe Mittelhaeuser, Raphael Bour, Brigitte L. Kieffer, Fabrice Riet, Emmanuel Darcq

**Affiliations:** ^1^Université de Strasbourg (UNISTRA), INSERM UMR-S 1329, Strasbourg Translational Neuroscience and Psychiatry, Centre de Recherche en Biomédecine de Strasbourg, Strasbourg, France; ^2^Unistra, CNRS, INSERM, CELPHEDIA, PHENOMIN-Institut Clinique de la Souris (PHENOMIN-ICS), Illkirch-Graffenstaden, France

**Keywords:** opioid, protracted abstinence, social deficits, withdrawal, morphine

## Abstract

**Introduction:**

Opioid use disorder (OUD) is a chronic relapsing condition caused by prolonged opioid exposure, which triggers adaptive changes in the brain. These changes make it challenging to control or abstain from consuming, and significantly increase the risk of relapse. While the physical symptoms of withdrawal typically resolve within a few days, extended abstinence is frequently accompanied by the progressive development of emotional disturbances. Additionally, abstinent individuals often report social disengagement, or even social isolation that worsen the condition and participates in the development of comorbidities. These disturbances are similarly observed in murine models of opioid abstinence.

**Methods:**

However, traditional methods for assessing social deficits in rodents often rely on simplistic paradigms with limited behavioral metrics. Here, we utilized a well-established model of morphine administration followed by protracted abstinence, combined with the Live Mouse Tracker (LMT) system. Using the real-time video-based automated LMT system, we conducted longitudinal recordings of social behaviors over a 4-week period of morphine abstinence, during repeated social interaction sessions.

**Results:**

The use of this method, offering an unbiased and precise behavioral characterization of social investigation between freely-moving male mice, revealed that while motor and activity-related disruptions emerge and resolve quickly immediately following the onset of abstinence, social deficits progressively intensify over time, reaching their peak 3 weeks after the final morphine administration. Additionally, the LMT provided detailed insights into subtle behavioral changes throughout the course of abstinence and within individual but also that early deficits in explorations and social interactions might serve as predictor for the severity of the late social deficits.

**Discussion:**

These results point out the need to improve and implement unbiased tracking methods for a deeper and refined understanding of rodent behaviors modeling psychiatric conditions.

## 1 Introduction

Opioid use disorder (OUD) is a psychiatric relapsing condition affecting ~16 million individuals globally and is thought to be the cause of 150,000 annual death around the world (Strang, 2020). Hence, OUD represents a significant global public health crisis and causes severe impairment and a low quality of life (Strang, 2020; Dydyk et al., 2025; Theisen et al., 2018). Regardless the reason of first use (i.e., for medical or recreational purpose), the primary cause for entering the OUD cycle is the repetition of opioid use that induces long-term adaptive changes in the brain (Leung et al., 2022; Lutz and Kieffer, 2013; Strang, 2020). Such alterations progressively high-jack brain circuits implicated in reward processing or emotion, eventually modifying the perception of hedonic stimuli, and constraint individual to focus their activity toward opioid use (Volkow and Blanco, 2021; Darcq and Kieffer, 2018). Furthermore, the repeated use triggers physiological modifications leading to tolerance and neuroadaptations which increase negative affect (Darcq and Kieffer, 2018).

Among others, an important drive for opioid recurrent use is the willingness to reduce withdrawal symptoms that quickly manifest after cessation (Pergolizzi et al., 2020). These symptoms are sub-categorized into physical and psychological symptoms. The physical signs of opioid withdrawal are temporary, starting quickly after the cessation of use and disappearing within few days, while the affective withdrawal symptoms, including dysphoria and anhedonia for instance, require more time to develop and may persist long after the opioid use cessation (weeks or even months). These long-lasting affective symptoms, also referred as hyperkatifeia (Shurman et al., 2010; Koob, 2021), are particularly debilitating and reduces drastically individuals’ life quality. These symptoms reflect the important neuroadaptation provoked by chronic opioid use but are yet to be fully understood. One important symptom reported by patients during protracted opioid abstinence is a profound feeling of social isolation, accompanied by an objective social disengagement (Galiza Soares et al., 2024). This isolation can exacerbate the affective symptoms already experienced during opioid abstinence (Ozdemir et al., 2023; Welsch et al., 2020; Galiza Soares et al., 2024; Lalanne et al., 2017), worsening the condition and favoring relapse. However, compared to the effort put into reducing physical withdrawal symptoms or anxiety and depression and despite preclinical studies (Lalanne et al., 2017; Lutz et al., 2014), helping OUD patients in regard to their reduction in sociability has not been not a priority. As a consequence, our understanding about the impact of opioid protracted abstinence on social behaviors remains poorly understood. Some insights came from animal models (Lutz et al., 2020; Welsch et al., 2020) and methodological aspects of these studies could be improved.

The social aspect of opioid withdrawal is often tested with paradigms consisting in observing when animals are close to a conspecific located in an enclosure, such as the three-chamber test (Kaidanovich-Beilin et al., 2011). These behavioral paradigms, although very informative, only relies on the binary choice between an object or a social stimulus. Furthermore, the experiment features that is extracted is commonly the presence of a subject in a specific zone even if the individuals are not objectively interacting. This experimental set-up can easily be challenging when treatments or specific conditions also affect locomotor activity; i.e., an animal could be immobile in the “interaction zone” without interacting with a conspecific. This is of importance, given the physical symptoms associated with opioid withdrawal that might interfere with the subjects’ motor capabilities. Hence, the classically used paradigms do not accurately represent social interactions that integrate a myriad of complex behaviors between conspecifics. Along with a simple nose-to-nose contact or a close presence near a conspecific, rodents also express approach behaviors, initiate and stop interactions that must be taken into consideration when evaluating social interactions and potential social deficits in animal models.

Using Live Mouse Tracker (de Chaumont et al., 2019) a real-time, video-based system for monitoring social behavior, we longitudinally analyzed activity-related as well as specific static and dynamic dyadic events (i.e., social interactions) between freely interacting mice in an open field over a 4-week period before and following escalating morphine administration. Such a model of passive opioid administration followed by deprivation has been used to evaluate opioid abstinence in murine models (Goeldner et al., 2011; Lutz et al., 2014; Lalanne et al., 2017; Welsch et al., 2020; Sourty et al., 2024). This methodological approach enabled us to dissect various facets of the murine social repertoire during protracted morphine abstinence. We identified subtle alterations in social behavior during morphine abstinence that refine our understanding of social alteration provoked by morphine abstinence. Our findings revealed a reduced exploration of the environment early after opioid cessation and lasting about 1 week, likely linked to the physical symptoms provoked by opioid cessation. More importantly, our results confirmed that protracted morphine abstinence induces social impairments, characterized by an incubation effect, as previously identified (Goeldner et al., 2011; Sourty et al., 2024; Welsch et al., 2023), without social alterations at the early stage of abstinence (24 h and 1 week following opioid cessation), indicating that these deficits evolve progressively and may evade detection by conventional behavioral assays. These results underscore the importance of employing unbiased, high-resolution behavioral tracking tools in preclinical neuroscience to more accurately characterize psychiatric disease models.

## 2 Methods

### 2.1 Animals

Adult male C57Bl6/J mice (>8 weeks old; Charles River) were housed in groups of 4–5 in a temperature and humidity-controlled room. All experiments were performed in accordance with the ARRIVE guidelines and regulations and were approved by the Regional Committee of Ethic in Animals Experiment Com’Eth (CE17, APAFIS #39888). One week before the first experiment a radio frequency identification (RFID) chip (APT12 PIT tags; Biomark, Inc., Boise) was subcutaneously implanted in each mouse. In that purpose, mice were quickly subjected to gas anesthesia (isofluorane) with local analgesia (Lidor 20 mg/mL, with 40 ul/10 g mouse) and then monitored (weight, well-being signs) during 1 recovery week.

### 2.2 Morphine treatment

According to a procedure previously described (Goeldner et al., 2011), escalating doses (20-100 mg/kg) of morphine (Laboratoires CDM Lavoisier) were administered intra-peritoneally twice daily for 5 days, followed by a single injection (100 mg/kg) the sixth day. Mice were then deprived from morphine, as a model of abstinence (Goeldner et al., 2011; Welsch et al., 2023; Sourty et al., 2024).

### 2.3 Experimental design

**Figure 1:**
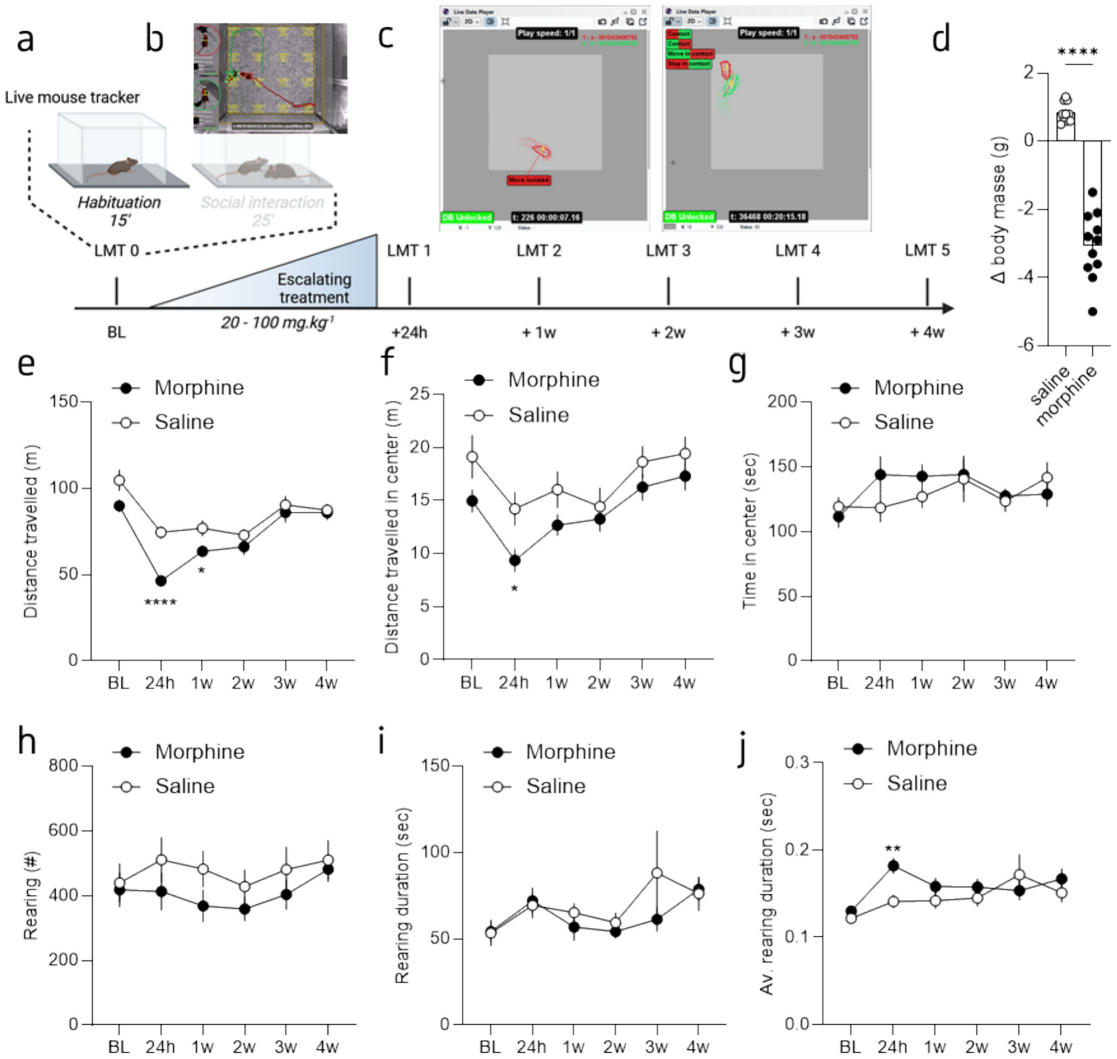
Morphine withdrawal induced a decrease of spontaneous exploration in absence of unfamiliar mouse. **(a)** Graphical representation of the behavioral design. Mice were recorded for 15 min (habituation) in an empty open field (OF) and then during a 25 min session of direct social interaction. Following a baseline (BL) session half of the mice received an escalating morphine treatment and then recorded in the same OF paradigm 24 h, one, two, three and for weeks post-treatment. **(b)** Screenshot of the LMT viewer [from de Chaumont et al. (2019)]. **(c)** Example of two LMT reconstructions, during habituation (left panel) and during social interaction (right panel). **(d)** Mean weight loss before and after the last day of treatment. **(e)** Mean distance traveled during the habituation phase for morphine- (brown) and saline-treated (grey) mice, across sessions. **(f)** Mean distance traveled in the center of the OF during the habituation phase for morphine- and saline-treated mice, across sessions. **(g)** Mean time spent in the center of the OF during the habituation phase for morphine- and saline-treated mice, across sessions. **(h)** Mean number of rearing during the habituation phase for morphine- and saline-treated mice, across sessions. **(i)** Mean rearing duration during the habituation phase for morphine- and saline-treated mice, across sessions. **(j)** Mean average rearing duration during the habituation phase for morphine- and saline-treated mice, across sessions. Data are represented as mean ± SEM. Morphine *n* = 11 and Saline control *n* = 10. *p < 0.05; ****p < 0.0001 for the comparison between saline- and morphine-treated mice.

The experimental design is shown in Figure 1. All experiments were conducted during the light phase, and mice within the same cage received the same treatment. Briefly, prior to any social interaction session, mice were allowed to freely explore the open-field (OF) for 15 min (habituation phase). Then, the focal mouse had the opportunity to interact with a conspecific (social interaction phase), of the same age and genetic background, placed in the OF for 25 min (Figure 1). All the sessions were recorded with the Live Mouse Tracker system (LMT) (de Chaumont et al., 2019) and analyzed with open source scripts from the lmt-analysis repository (https://github.com/fdechaumont/lmt-analysis; Figure 1 and Supplementary Figure S1). A total of 5 sessions were performed. In a first session (session 0), we measured the basal levels of activity and social behaviors of each mouse with the LMT system. The week after, a group of mice received the escalating morphine treatment (Figure 1); while control animals receive an injection of saline solution. We then repeated the behavioral paradigms and evaluated the activity and social interactions 24 h after the last injection (session 1) and then once a week, over 4 weeks (session 2 to 5).

#### 2.3.1 Habituation

Mice were tested individually in an of 50 × 50 cm filled with sawdust (~1.5 cm). At the beginning of the test, the individual was placed at the periphery of the arena and then allowed to freely explore the arena for 15 min. The distance traveled, rearing events, time and distance in the center of the OF were recorded during the session.

#### 2.3.2 Social interactions with an unfamiliar individual

At the end of the habituation phase, an unfamiliar newcomer of the same age and genetic background was placed in the arena and the social behaviors of each individual were recorded for 25 min. For each session, a novel unfamiliar newcomer was introduced to ensure that social interactions were not repeated with the same individual.

### 2.4 Live mouse tracker data analysis

In addition to the location of the animal, for the calculation of the distance and time in specific zones of the OF and movements (rearing), the data were analyzed using custom made Python scripts available on GitHub.^1^ Individuals’ behaviors were first rebuilt using the Rebuild_All_Events.py script (in LMT/scripts/ folder), then different social behavioral measures were extracted thanks to the computeMeasuresIdentityProfileOneMouseAutomatic.py script (in LMT/scripts/ folder). In this study, we extracted dyadic events dissociated by their dynamic or static aspect. Here, based on de Chaumont et al. (2019) we evaluated static dyadic events consisting in moves, stops and contacts with the newcomer. In regard to the contacts, side by side, nose to nose and nose to anogenital area contacts were evaluated. Individuals are considered “in movement” when the focal animal is moving (speed > 5 m/s) while expressing the behavior of interest.

For the dynamic dyadic events, we extracted several behaviors. The approaches, defined as the focal animal getting closer to the newcomer within a circular zone of 2 body lengths and then making or not contact. The breaks, defined as the focal animal is getting away (higher speed) from the other individual it has been in contact with; the speed of the focal animal is higher than the speed of the other animal. Finally, we evaluated when the mouse followed the newcomer, defined as the focal animal is walking in the path of the other individual. In this specific case, the two animals are moving at a speed >5 cm/s, the angles between the two animals are less than 45° apart, and the mass centre of the follower (the focal animal) is within a follow zone of one mean body length of width and two mean body lengths of length.

### 2.5 Statistics

**Figure 2:**
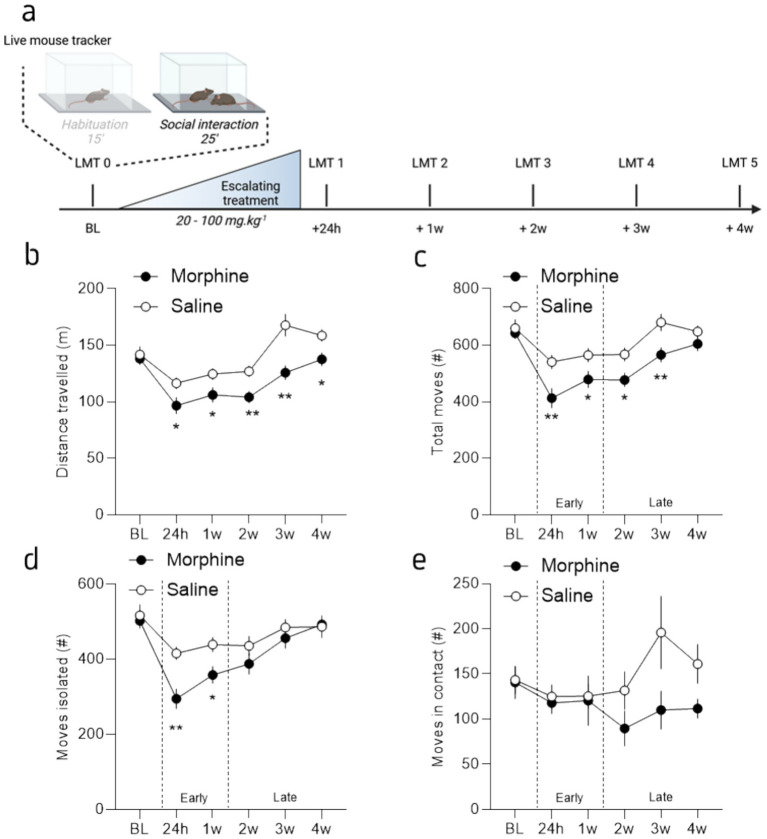
Protracted morphine decreased social induced activity behaviors differentially over time. **(a)** Graphical representation of the behavioral design. **(b)** Mean distance traveled during the interaction phase for morphine- (brown) and saline-treated (grey) mice, across sessions. **(c)** Mean number of total moves (animal moving at a speed >5 cm/s) during the interaction phase for morphine- and saline-treated mice, across sessions. **(d)** Mean number of total moves while isolated during the interaction phase for morphine- and saline-treated mice, across sessions. **(e)** Mean number of total moves in contact with the interactor during the interaction phase for morphine- and saline-treated mice, across sessions. Data are represented as mean ± SEM. Morphine *n* = 11 and Saline control *n* = 10. *p<0.05; **p<0.01 for the comparison between saline- and morphine-treated mice.

Data are presented as means ± SEM with corresponding dot plots overlaid and were analyzed using Welsh’s *t*-test, one or two-way ANOVAs when appropriate, with sessions as repeated measures. Significant main effects (*p* ≤ 0.05) were followed by uncorrected Fisher’s LSD or Tukey’s multiple comparisons tests. We used Tukey’s test when comparing all group means, as it provides a conservative control of type I error in multiple comparisons. In contrast, Fisher’s LSD test was applied in cases with a small number of comparisons (see Figure 2) and when specific *a priori* hypotheses were tested, as it offers higher statistical power. *Z*-score values were calculated for each mouse for each variable, on each session, using the following formula: *Z* = (X−*μ*)/*σ* where X represents the individual data for the observed parameter while μ and σ represent the mean and standard deviation of the control group (saline-treated mice during the baseline session). Finally, for early (sessions 1 and 2) and late (session 3 to 5) phases, *Z*-score values for each session were added and divided by the number of session as follow: early values: [(*Z*-score_session1_ + *Z*-score_session2_)/2]; late values: [(*Z*-score_session3_ + *Z*-score_session4_ + *Z*-score_session5_)/3]. Finally, the combined social Z-score was obtained by averaging each individual *Z*-score of the number of contacts, social approaches and dyadic events and the combined motor *Z*-score was obtained by averaging each individual *Z*-score of the total distance traveled, the distance traveled in the center and the number of rearing. All data was analyzed using GraphPad Prism (v10) software.

## 3 Results

### 3.1 Morphine abstinence reduced transiently spontaneous exploration in the absence of interactor

To test the impact of morphine abstinence on spontaneous exploration, we measured exploration during an initial 15 min session of open field (OF). Then to evaluate the impact of morphine abstinence on social behaviors, mice were allowed to freely interact for 25 min with an unfamiliar counterpart (Figure 1 and Supplementary Figure S1). To see the evolution of behavioral phenotype, these measures were done before (baseline or session 0) and 24 h (session 1), 1 week (session 2), 2 weeks (session 3), 3 weeks (session 4) and 4 weeks (session 5) after chronic morphine treatment, during the subsequent withdrawal period. Specifically, after the session 0 (baseline), half of the mice underwent an escalating morphine treatment (doses 20-100 mg/kg; i.p.); the other half receiving saline injection (i.p.) (Figure 1a). As previously reported (Goeldner et al., 2011), this treatment led to a substantial weight loss when we compared the weight before and after the last day of treatment (Figure 1d; Welsh’s t-test: *F*_(10,9)_ = 12.17; *p* < 0,001). Subsequently, mice were subjected to five additional OF sessions: the first session 24 h after the final morphine injection, followed by weekly sessions over a 4-week period (Figure 1a). Each session was recorded and analyzed using the LMT system to assess activity-related and social behaviors.

Our behavioral design allows us to first compare the spontaneous exploration of each habituation session, without a social interactor. The analysis of the total distance traveled in the OF revealed a drug and session effect as well as an interaction (Figure 1e; 2-way Repeated Measure (RM) ANOVA; main effect of drug: *F*_(1, 19)_ = 7.83 *p* = 0.011; main effect of session: *F*_(3.27, 62.14)_ = 34.57, *p* < 0.0001; interaction drug x session: *F*_(5, 95)_ = 4.07, *p* = 0.002). The Tukey’s post-hoc analysis showed that, while the baseline OF (prior morphine treatment) was similar between saline and morphine treated mice (*p* = 0.052), session 1 and 2 (24 h and 1 week after the last injection) were different between the two groups (session 1: *p* > 0.0001; session 2: *p* = 0.0268); no other difference was observed (*p* = 0.24; *p* = 0.58; *p* = 0.78; for session 3, 4 and 5, respectively). A closer analysis of the distance traveled in the most anxiogenic part of the OF, the center, did not reveal drug (Figure 1f; 2-way RM ANOVA; main effect of drug: *F*_(1, 19)_ = 4.2, *p* = 0.054) nor drug x session interaction significant effects (2-way RM ANOVA; interaction drug x session: *F*_(5, 95)_ = 0.84, *p* = 0.50) but a main effect of session (2-way RM ANOVA: *F*_(3.410, 64.80)_ = 11.54, *p* < 0.0001). The Tukey’s post-hoc analysis revealed a difference between the groups for session 1 (24 h post-treatment; *p* = 0.02), the analysis of the percentage of time spent in the center showed no significant effects (Figure 1g; 2-way RM ANOVA; main effect of drug: *F*_(1, 19)_ = 0.31, *p* = 0.58; main effect of session: *F*_(3.79, 72.17)_ = 2.10, *p* = 0.09; interaction drug x session: *F*_(5, 95)_ = 1.27, *p* = 0.28). Furthermore, although a tendency toward a reduction, the analysis of rearing behavior, considered as part of the exploratory behaviors (Lever et al., 2006), showed no specific difference between groups. Indeed, the total number of rearing over the 15 min is similar between groups across the sessions (Figure 1h; 2-way RM ANOVA; main effect of drug: *F*_(1, 19)_ = 1.03, *p* = 0.32; main effect of session: *F*_(2.75, 52.42)_ = 3.10, *p* = 0.04; interaction drug x session: *F*_(2.76, 52.42)_ = 0.88, *p* = 0.45). Similarly, the total duration spent rearing is similar between the two groups (Figure 1i; 2-way RM ANOVA; main effect of drug: *F*_(1, 19)_ = 0.46, *p* = 0.51; main effect of session: *F*_(1.95, 36.97)_ = 3.11, *p* = 0.06; interaction drug x session: *F*_(1.95, 36.97)_ = 1.01, *p* = 0.37). Interestingly, the tendency toward a reduced number of rearing combined with a similar time rearing led to a tendency toward an increased average time spent rearing (Figure 1j; 2-way RM ANOVA; drug: F_(1, 19)_ = 1.33, *p* = 0.26; main effect of session: *F*_(2.15, 40.95)_ = 5.65, *p* = 0.01; interaction drug x session: F_(2.15, 40.95)_ = 2.69, *p* = 0.08). The Tukey’s post-hoc analysis revealed a difference between the two groups only at 24 h post-treatment (*p* = 0.002), with a greater time for the morphine-treated mice. These findings reveal that a reduction of the ambulatory behavior in a familiar environment without interactor is present 24 h after the stop of morphine treatment. However, it is not accompanied with strong anxiety-related traits. We only observed a reduced distance traveled in the center and a tendency to rear for a longer time at the earliest time point (i.e., 24 h). These exploratory alterations are likely the consequences of physical and anxiogenic withdrawal symptoms present during the first days of opioid withdrawal.

### 3.2 Morphine abstinence decreased exploration persistently in the presence of a conspecific

**Figure 3:**
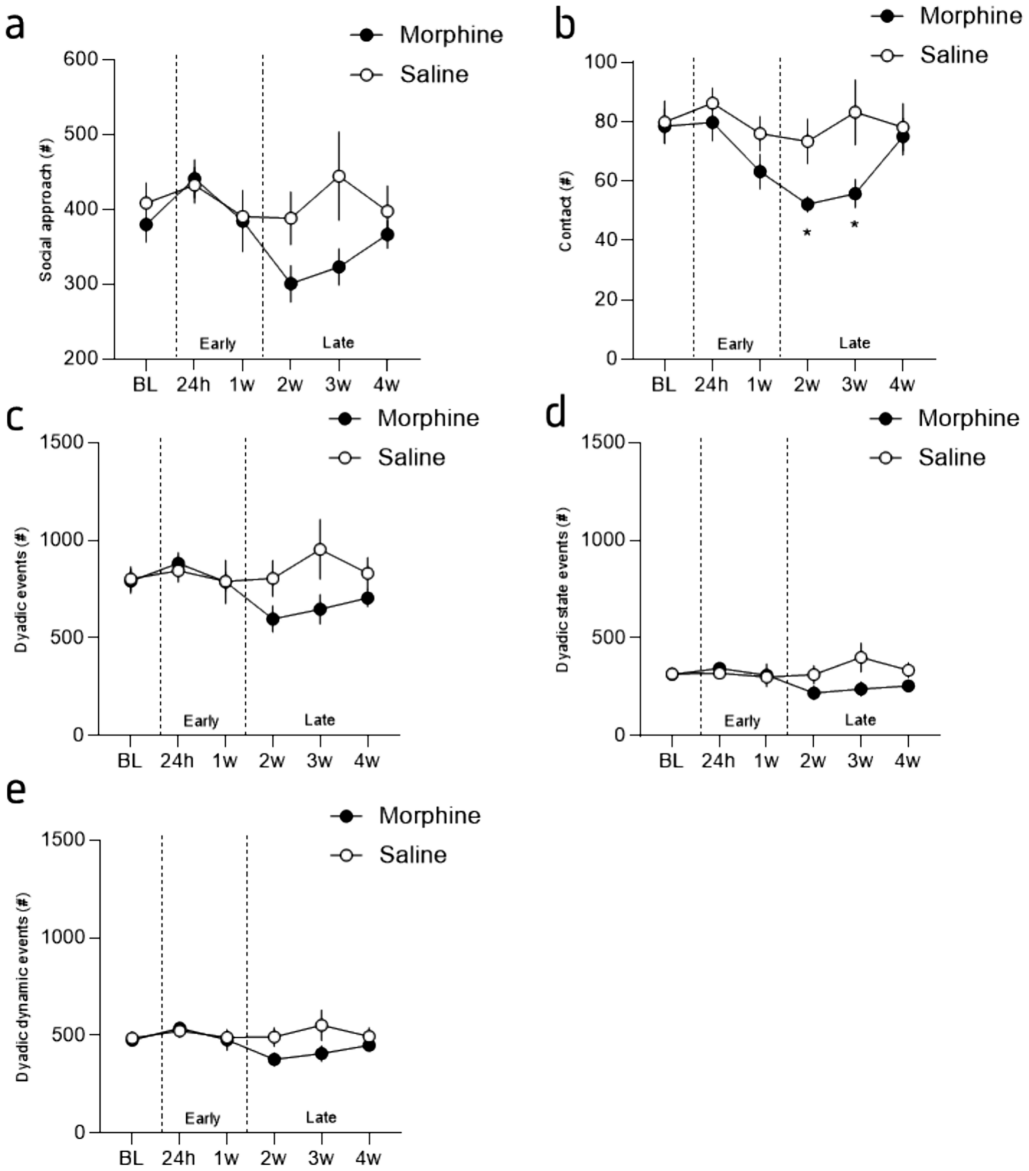
Incubation of the social deficit induced by morphine protracted abstinence. **(a)** Mean number of social approaches during the interaction phase for morphine- (brown) and saline-treated (grey) mice, across sessions. **(b)** Mean number of contacts during the interaction phase for morphine- and saline-treated mice, across sessions. **(c)** Mean number of dyadic events during the interaction phase for morphine- and saline-treated mice, across sessions. **(d)** Mean number of dyadic state events during the interaction phase for morphine- and saline-treated mice, across sessions. **(e)** Mean number of dyadic dynamic events during the interaction phase for morphine- and saline-treated mice, across sessions. Data are represented as mean ± SEM. Morphine *n* = 11 and Saline control *n* = 10. *p < 0.05 for the comparison between saline- and morphine-treated mice.

The analysis of the 25 min social interaction OF sessions revealed a different effect of morphine abstinence over activity when an interactor is present. Similar to the exploration without social interactor, the statistical analysis revealed a reduction of the total distance traveled by morphine abstinent mice (Figure 3b; 2-way RM ANOVA; main effect of drug: *F*_(1, 19)_ = 11.81, *p* = 0.003; main effect of session: *F*_(3.852,73.18)_ = 28.99, *p* < 0.0001; interaction drug x session: *F*_(5, 95)_ = 3.27, *p* = 0.009). Here, the Tukey’s post-hoc analysis between groups showed that all the sessions, at the exception of the baseline session (*p* = 0.68), are different between morphine and saline treated mice (*p* = 0.04; p = 0.04; *p* = 0.004; p = 0.002; p = 0.01; for session 1 to 5, respectively). In accordance with the distance traveled, the statistical analysis of moves, defined as the animal moving at a speed >5 cm/s showed a session and a drug effect (Figure 3c; 2-way RM ANOVA; main effect of session: *F*_(4.10, 77.96)_ = 24.92, p < 0.0001; drug: *F*_(1, 19)_ = 9.41, *p* = 0.006) but no drug x session interaction (2-way ANOVA; drug x session: *F*_(5, 95)_ = 2.14, *p* = 0.07); with a difference between groups for all the timepoints, at the exception of the session 0 and 5 (Tukey’s post-hoc analysis: *p* = 0.64, *p* = 0.007, *p* = 0.036, *p* = 0.015, p = 0.009 and *p* = 0.19 for each session, from 0 to 5 respectively). Together, these results suggest that, beyond the temporary early effects of morphine withdrawal observed during habituation, a long-lasting change in behavior occurs when protracted-abstinent mice are in the presence of a conspecific. This pattern can be viewed as a biphasic effect of opioid abstinence: an initial, early, and transient reduction in ambulatory behavior, likely driven by physical symptoms, that leads to reduced exploration during both OF habituation (without an interactor) and when social interaction is available; followed by a second, progressive effect characterized by reduced exploration only when social interaction is possible, indicating a gradual alteration in behavior due to social deficits.

### 3.3 Social deficits incubate along protracted abstinence

We took advantage of the LMT capacity to analyze the animals’ movements and dissociated those made by the animals when it is alone or in contact with an unfamiliar mouse. This closer analysis revealed that the reduction in moves observed in abstinent mice during the first week post-treatment (i.e., session 24 h and 1 week; early phase) is mostly driven by a reduction of the moves made by the animal when they are isolated, without any modification of the moves made in contact (Figures 3d,e). On the contrary, the reduction of the total moves observed after the first week post-treatment (from 2 to 4 weeks post-treatment; late phase) is mostly due to a reduction of the moves made in contact with the interactor (Figures 3d,e). Indeed, the statistical analysis showed a session and drug x session effect but no drug effect for the moves isolated (Figure 3d; 2-way RM ANOVA; main effect of drug: *F*_(1, 19)_ = 4.24, *p* = 0.054; main effect of session: *F*_(3.612, 68.63)_ = 19.01, *p* < 0.0001; drug x session: *F*_(5, 95)_ = 2.884, *p* = 0.0181) and only a drug x session interaction for the moves in contact (Figure 3e; 2-way RM ANOVA; main effect of drug: *F*_(1, 19)_ = 2.20, *p* = 0.15; main effect of session: *F*_(3.16, 59.01)_ = 2.02, *p* = 0.12; drug x session: *F*_(5, 95)_ = 2.20, *p* = 0.049). These results seem to confirm the biphasic effect of morphine abstinence, with a rapid and transient general reduction of activity lasting ~1 week and that is observable when individuals are isolated in the OF (Figures 1b, 3b). Furthermore, this general reduction of activity is accompanied with a slight increase in anxiety (reduced distance traveled in the center; Figure 1c). Then, within ~2–3 weeks this alteration seems to reverse, with an alteration of the activity only when a social interaction is possible. Importantly, that effect is not attributable to a reduction of the contacts nor the social approaches made by the interactors (see Supplementary Figure S1).

**Figure 4:**
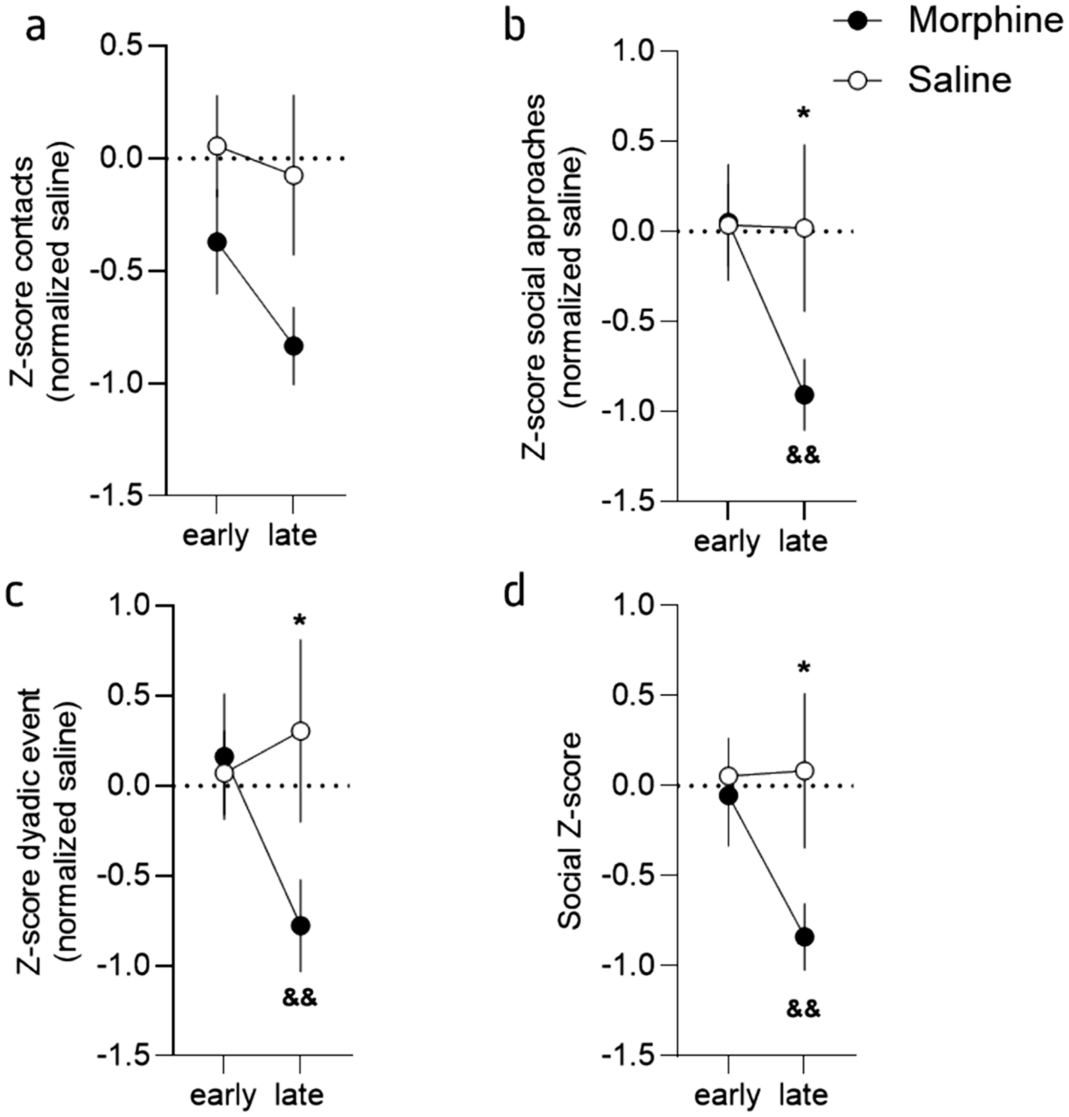
Protracted morphine decreased social interaction at a late stage but not early. **(a)** Mean *Z*-score (normalized to the saline mice) for the contacts for morphine- (brown) and saline-treated (grey) mice, at the early (24 h to 1 week) and late (2 to 4 weeks) stages. **(b)** *Z*-score (normalized to the saline mice) for the social approaches for morphine- and salinetreated mice, at the early and late stages. **(c)** Mean *Z*-score (normalized to the saline mice) for the dyadic events for morphine- and saline-treated mice, at the early and late stages. **(d)** Mean combined *Z*-score (normalized to the saline mice) for morphine- and saline-treated mice, at the early and late stages. This *Z*-score was obtained by averaging each individual *Z*-score of the number of contacts, social approaches and dyadic events. Data are represented as mean ± SEM. Morphine *n* = 11 and Saline control *n* = 10. *p < 0.05 for the comparison between saline- and morphine-treated mice; &&*p* < 0.01 for the comparison between the early and late phase for the morphine-treated mice.

Based on these findings, we further extracted dyadic events (de Chaumont et al., 2019), which refers to the patterns of behavior that occur between two individuals in a relationship or social context. These events include all the behaviors made in contact with the conspecific such as moves, stops and rearing as well as side-by-side, nose-to-nose and nose-to-anogenital part contacts (see Supplementary Figure S2). Furthermore, LMT can extract dynamic social events such as approach with or without making contact, follows (i.e., when an individual is walking in the path of another individual) and breaking contacts. These analyses confirmed our previous observations with no alterations during the early stage (24 h and 1 week after opioid cessation) but a reduced social repertoire after 2 weeks of opioid cessation. More precisely, in accordance with our previous results, the statistical analysis of the number of social approaches revealed a significant session effect and a treatment x session interaction but no drug effect (Figure 4a; 2-way RM ANOVA; main effect of drug: *F*_(1, 19)_ = 1.81, *p* = 0.19; main effect of session: *F*_(3.544, 67.34)_ = 3.28, *p* = 0.02; interaction drug x session: *F*_(5, 95)_ = 2.35, *p* = 0.046). In a similar manner, the statistical analysis of the occurrence of all the contacts revealed a significant session effect and a treatment x session interaction but no drug effect (Figure 4b; 2-way RM ANOVA; main effect of drug: *F*_(1, 19)_ = 2.85, *p* = 0.11; main effect of session: *F*_(3.97, 75.47)_ = 5.42, *p* = 0.0007; interaction drug x session: *F*_(5, 95)_ = 2.64, *p* = 0.03). The Tukey’s post-hoc analysis between groups revealed a significant difference between groups for session 3 (2 weeks post morphine treatment; *p* = 0.02) and 4 (3 weeks post morphine treatment; *p* = 0.0404) but not the others (min *p* value = 0.14). Moreover, a closest analysis of the type of contact that is modified by morphine abstinence revealed that the reduction in contact cannot be explained by a specific reduction in oro-genital or oro-oral contacts (see Supplementary Figure S2) but likely a more complex and global alteration of the social behavior repertoire.

In that regard, the 2-way ANOVA analysis for the total dyadic events (de Chaumont et al., 2019) during the whole interaction across sessions suggests that these events are mildly impacted by protracted morphine abstinence (Figures 4a,e; 2-way RM ANOVA; main effect of drug: *F*_(1, 19)_ = 1.52, *p* = 0.23; main effect of session: *F*_(3.33, 63.33)_ = 1.51, *p* = 0.22; interaction drug x session: F_(5, 95)_ = 2.484, *p* = 0.0369). Although the Tukey’s post-hoc analysis revealed a tendency for session 3 and 4 (2- and 3-weeks post morphine treatment, p = 0.0872 and p = 0.0946 respectively), no significant differences were observed (*p* > 0.20 for all the other comparisons). The closer analysis of dynamic and state dyadic events [see methods 2.4 and de Chaumont et al. (2019)], revealed the same pattern (Figure 4d; 2-way RM ANOVA; main effect of drug: *F*_(1, 19)_ = 1.15, p = 0.23; main effect of session: *F*_(3.27, 62.21)_ = 1.22, *p* = 0.31; interaction drug x session: *F*_(5, 95)_ = 2.99, *p* = 0.01) while no difference at all were observed for the dynamic dyadic events (Figure 4e; 2-way RM ANOVA; main effect of drug: F_(1, 19)_ = 1.45, *p* = 0.24; main effect of session: *F*_(3.44, 65.37)_ = 1.84, *p* = 0.14; interaction drug x session: F_(5, 95)_ = 2.00, *p* = 0.09).

Together, these results demonstrated that morphine withdrawal provokes important alterations in exploration and activity during the first hours of opioid cessations, an effect that lasts during protracted abstinence and that are accompanied with social deficits. In order to refine the analyze of these findings and better highlight the differences observed during the early (i.e., 24 h and 1 week) and the later phases (i.e., from week 2 to 4), we calculated “social *Z*-scores” (Guilloux et al., 2011) for the number of contacts, social approaches and total dyadic events (see Supplementary Figure S3) and then calculated *Z*-scores for the early (24 h and Week 1) and late (week 2 to 4) phase of abstinence (see Supplementary Figure S3). This visualization of the data, that standardizes raw scores to saline values at baseline and allows for comparisons across different measures and time, clearly showed that morphine treated mice modify their social behavior during the late phase but not the early one (Figure 2). Indeed, at the exception of the contact (Figure 2a; 2-way ANOVA; main effect of drug: *F*_(1, 19)_ = 3.30, *p* = 0.08; main effect of phase: *F*_(1, 19)_ = 4.25, *p* = 0.05; interaction drug x phase: *F*_(1, 19)_ = 1.36, *p* = 0.26), the analysis revealed a significant difference between groups and for the two different phases for these variables (Figures 2b,c; social approaches: 2-way ANOVA; main effect of drug: *F*_(1, 19)_ = 1.27, *p* = 0.27; main effect of phase: *F*_(1, 19)_ = 2.48, *p* = 0.13; interaction drug x phase: *F*_(1, 19)_ = 6.89, *p* = 0.02; dyadic events: 2-way ANOVA: main effect of drug: *F*_(1, 19)_ = 1.31, p = 0.27; main effect of phase: *F*_(1, 19)_ = 5.69, *p* = 0.03; interaction drug x phase: *F*_(1, 19)_ = 5.32, *p* = 0.03). The LSD post-hoc analysis revealed the same pattern for all the comparisons with a difference at the late phase between groups (social approaches: *p* = 0.04, dyadic events: *p* = 0.05) and a difference between the phase for the morphine treated mice (social approaches: *p* = 0.01, dyadic events: *p* = 0.003). This suggest that most of the social deficits develop progressively and are expressed during the late phase. In accordance, the combined social *Z*-score (Guilloux et al., 2011), calculated based on the three variables, for the early and late phases revealed a delayed effect of morphine abstinence (Figure 2d; 2-way ANOVA: main effect of drug: *F*_(1, 19)_ = 1.99, *p* = 0.17; main effect of phase: *F*_(1, 19)_ = 4.37, *p* = 0.05; interaction drug x phase: *F*_(1, 19)_ = 5.08, *p* = 0.04). The Fisher LSD post-hoc analysis between groups revealed a significant difference between groups during the late (*p* = 0.03) but not the early (*p* = 0.79) phase and a phase effect for the morphine group only (*p* = 0.005; saline treated mice: *p* = 0.91).

### 3.4 Morphine treatment consequence on body weight as well as early motor deficits have a predictive value for social deficits provoked by morphine abstinence

**Figure 5:**
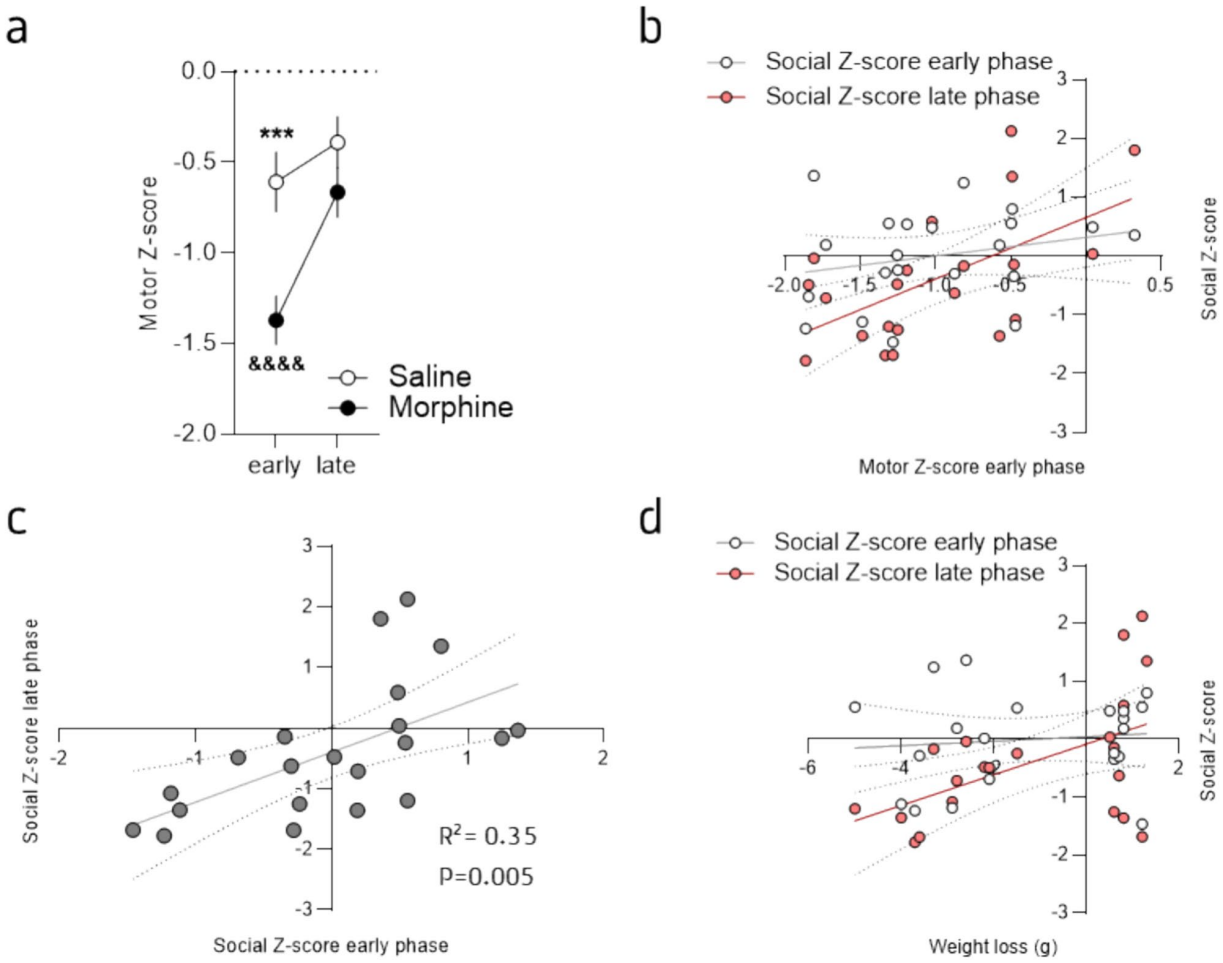
Early motor deficits predict social deficits induced by morphine protracted abstinence. **(a)** Mean *Z*-score for the “motor” variables analyzed during the OF habituation sessions for morphine- (brown) and saline-treated (grey) mice. Data are represented as mean ± SEM. ***p < 0.001 for the comparison between saline- and morphine-treated mice; &&&&*p* < 0.0001 for the comparison between the early and late phase for the morphine-treated mice. **(b)** Linear regression showing a positive correlation between the early motor deficits (motor *Z*-score for the early phase) and the social *Z*-score at late (*r*^2^ = 0.33, *p* = 0.007; red circles) but not early stage (*r*^2^ = 0.06, *p* = 0.29; white circles). **(c)** Linear regression demonstrating the positive correlation between the social *Z*-scores at early and late stages (*r*^2^ = 0.35, *p* = 0.005). **(d)** Linear regression showing a positive correlation between the weight loss during morphine treatment and the social *Z*-score at late (*r*^2^ = 0.26, *p* = 0.02; red circles) but not early stage (*r*^2^ = 0.01, *p* = 0.66; white circles). Morphine *n* = 11 and Saline control *n* = 10.

Based on our results we then sought to evaluate if the early deficits in exploration (Figure 1) might predict the social deficits (Figures 4a–c), and so could be used as a prediction factor. Hence, first, we calculated a “motor *Z*-score,” that combines the *Z*-score values of the total distance traveled, the distance traveled in the center of the OF and the number of rearing during the 15 min session of habituation, for each animal and each session (see Supplementary Figure S4), that likely reflect the physical alterations of opioid cessation. The combined *Z*-score of all these individual behaviors was then calculated to represent the global exploratory and motor alterations induced by morphine withdrawal and abstinence. As expected, we observed the opposite effect compared to the social deficits previously described (Figure 2), with a strongly reduced value for morphine-treated mice but only for the early phase of withdrawal (Figure 5a; 2-way ANOVA: main effect of drug: *F*_(1, 19)_ = 7.83, *p* = 0.01; main effect of session: *F*_(1, 19)_ = 28.64, *p* < 0.0001; interaction drug x session: F_(1, 19)_ = 7.98, p = 0.01). We then sought to correlate the exploratory/motor alterations with the social deficits. To do so we evaluated the potential correlation between the motor *Z*-score of the early phase with the *Z*-scores for the social deficits for both the early and late phases (Figure 5b). This analysis revealed that the early motor *Z*-score correlates with the late *Z*-score for the social deficits but not the early one (Figure 5b; early: *r*^2^ = 0.06; *F*_(1, 19)_ = 1.18, *p* = 0.29; late: *r*^2^ = 0.33; *F*_(1, 19)_ = 9.30, *p* = 0.01). Furthermore, the analysis between the early and late social *Z*-scores also revealed a positive correlation (Figure 5c; *r*^2^ = 0.35; *F*_(1, 19)_ = 10.23, *p* = 0.01). In addition, based on a drastic effect of morphine on body mass during the escalating treatment (Figure 1b) we also evaluated whether this morphine-induced body mass loss could predict the social deficits. This analysis also revealed a positive correlation for the late social deficits (late *Z*-score, Figure 5d; *r*^2^ = 0.26; *F*_(1, 19)_ = 6.59, *p* = 0.02) but not the early one (Figure 5d; *r*^2^ = 0.01; *F*_(1, 19)_ = 0.2, *p* = 0.66). Nonetheless, the natural (baseline) social behavior also seems to correlate with late social deficits for morphine abstinent mice (see Supplementary Figure S4). These findings suggest that, although natural social traits are important to consider, the physical impact of morphine treatment (i.e., weight loss), the early motor deficits, that likely represent a proxy of the physical withdrawal symptoms, and the early social deficits, all might serve as predictor for the severity of the social deficits expressed at a later phase.

## 4 Discussion

This study demonstrates that opioid abstinence induces social impairments, which unfold in a temporally dynamic, biphasic manner consistent with an incubation process. We first observed a rapid and transient disruption of exploratory behaviors (e.g., total distance traveled, time spent in the center of the open field, and rearing), independent of social interaction. These alterations emerged as early as 24 h after morphine withdrawal and persisted for approximately 1 week, aligning with the well-described physical withdrawal symptoms associated with opioid abstinence (Welsch et al., 2020; Ozdemir et al., 2023). Although these disturbances resolved quickly in the absence of an interactor, they remained detectable weeks after the end of the treatment in the presence of a social interactor. This pattern suggests that opioid withdrawal initially induces a generalized behavioral disturbance, likely reflecting acute physical withdrawal (Pergolizzi et al., 2020; Ozdemir et al., 2023; Kalamarides et al., 2024), which gradually gives way to alterations specifically affecting social behavior. Interestingly, during this early phase (24 h to 1-week post-treatment), no clear deficits in social behavior were observed. Instead, morphine-treated mice reduced their moves in isolation but not when a conspecific was present, as previously described (Stickney and Morgan, 2021). This may indicate that withdrawal-induced negative affect transiently promotes prosocial behaviors as a compensatory response.

At later stages, between 2- and 4-weeks post-treatment, animals exhibited pronounced social deficits that resolved by four weeks, consistent with prior reports (Pomrenze et al., 2022; Goeldner et al., 2011). Unlike classical social interaction assays, the LMT paradigm enabled a multidimensional assessment of social behaviors (de Chaumont et al., 2019), revealing a robust reduction in the number of contacts and a trend toward fewer social approaches and dyadic events during this late abstinence phase. However, no changes were detected in specific interaction subtypes (e.g., oral–oral or oral–genital contacts, static or dynamic dyadic events), suggesting that opioid withdrawal broadly disrupts the social repertoire rather than selectively targeting particular behavioral domains. Standardization of outcomes using *Z*-scores (Guilloux et al., 2011) confirmed this biphasic profile: an early, generalized behavioral disruption followed by a delayed but more specific impairment of social behaviors. This trajectory is consistent with previous evidence for an incubation of social deficits (Galiza Soares et al., 2024; Goeldner et al., 2011), potentially involving alteration in serotonergic mechanisms (Lalanne et al., 2017; Pomrenze et al., 2022).

To further investigate individual variability, we assessed whether early general deficits could predict later social impairments. Our analyses revealed that the extent of exploratory disruption during the early phase correlated with the subsequent social deficits. In addition, social behavior scores were positively correlated between early and late phases. These findings suggest that early signs of discomfort or withdrawal-related behavioral disturbances may serve as predictors of later-emerging social impairments. Furthermore, our analysis showed that weight loss provoked by the morphine treatment also correlate with the late social deficits. At the preclinical level, this underscores the importance of characterizing phenotypic traits to better understand the trajectory of opioid withdrawal, as it was identified with impulsivity (Belin et al., 2008) or with morphine reward effect (Pomrenze et al., 2022). Clinically, these results highlight the potential value of evaluating patients’ social skills and baseline “social state” prior to initiating chronic opioid treatment, in order to anticipate long-term side effects. Moreover, standardized assessments of early withdrawal symptoms could provide a predictive tool to identify individuals at greater risk of developing persistent social deficits, thereby informing more personalized treatment and care strategies.

Together, our findings highlight the predictive value of early behavioral alterations for later-emerging social impairments. Nonetheless, several limitations should be acknowledged. First, the present study employed a passive administration protocol (i.p. injections of escalating doses of morphine). Future work should confirm these results in paradigms allowing voluntary opioid intake, which more closely models human patterns of drug use. Moreover, in the context of opioids, it is crucial to consider the initial motivation for drug consumption when evaluating abstinence profiles. The long-term consequences are likely to differ between individuals using opioids for pain management versus those engaging in recreational use. Second, due to technical constraints, animals were monitored only during the open field sessions. While this approach allowed for a precise characterization of exploratory and social behaviors, continuous tracking within the LMT system before, during, and after morphine exposure would offer a more comprehensive understanding of the dynamics of opioid use and abstinence. In addition, integrating assessments of other phenotypic traits, such as impulsivity or risk-taking (Belin et al., 2008) and vocalization recording (de Chaumont et al., 2021), with LMT tracking could yield clearer and more accurate predictive insights into abstinence trajectories. Finally, the study was conducted exclusively in male mice, precluding the evaluation of sex differences. Given accumulating evidence for sex-specific vulnerability and trajectories in substance use disorders (Nicolas et al., 2022; Becker and Koob, 2016; Becker, 2016; Roth et al., 2004), future studies should incorporate both sexes to better capture interindividual variability.

Taken together, these findings, while requiring further validation, confirmed the critical impact of opioid abstinence on social behaviors and underscore the importance of early behavioral markers as predictors of later social impairments and highlight potential avenues for improving the clinical management of opioid withdrawal.

## Data Availability

The raw data supporting the conclusions of this article will be made available by the authors, without undue reservation. Publicly available datasets can be found here: https://zenodo.org/records/17251674 and https://doi.org/10.5281/zenodo.17251674.

## Footnotes

^1^ https://github.com/fdechaumont/lmt-analysis/releases/tag/v1.0.5
