# Supplementary material for: Incubation of social deficit during morphine abstinence in male mice using a novel unbiased and automatized method
Source: Front Behav Neurosci. 2025 Oct 29;19:1697469. doi: 10.3389/fnbeh.2025.1697469 (PMC12605237; doi:10.3389/fnbeh.2025.1697469)
Supplement: Supplementary file 1 [file Data_Sheet_1.PDF]

Supplementary Material

1 Supplementary Figures and Tables

Figure S1: LMT trajectories & number of contacts made by interactors, across time.

Figure S2: Mean static and dynamics dyadic events, across time.

Figure S3: Dyadic, contacts and social approaches Z-score across time.

Figure S4: Motor Z-scores & correlation baseline vs early and late social Z-scores.

1.1 Supplementary Figures

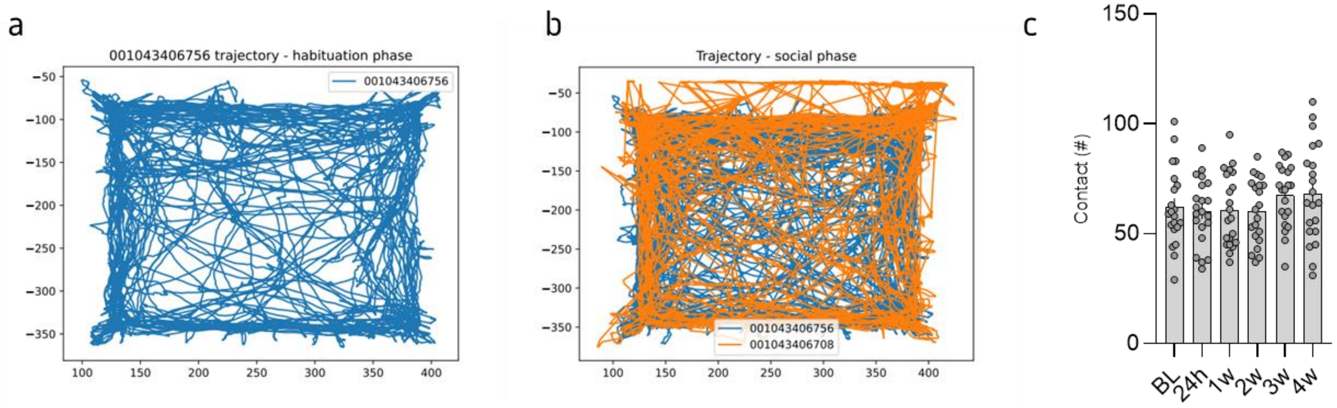

Supplementary Figure 1. a) Example of one mouse trajectory reconstructed based on the LMT video recording for a single mouse, during habituation. b) Example of two mice trajectories reconstructed based on the LMT video recording for a single mouse, during social interaction. **c**) Number of contacts made by the interactors during the LMT sessions, across time. No specific difference was observed across the LMT sessions (1-way ANOVA: session: $F_{(3.368,73.16)}=1.231$, $p=0.305$). Data are represented as mean ± SEM and individual values are plotted (grey circles), n=21.

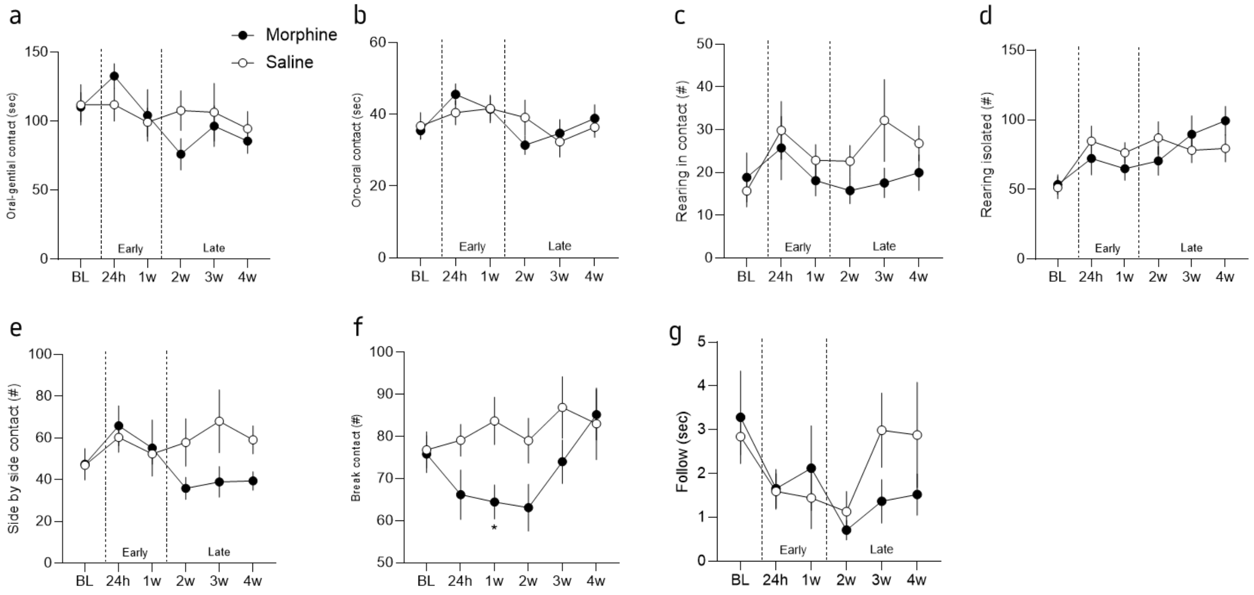

Supplementary Figure 2. a) Mean duration of Oral-genital contacts for the morphine- (brown) and saline- (grey) treated mice, across LMT sessions. No difference between the two treatments was observed (2-way ANOVA: session: $F_{(3.313,62.95)}$=2.852, p=0.039; drug: $F_{(1,19)}$=0.099, p=0.757; drug x session: $F_{(3.313,62.95)}$=1.482, p=0.225). **b**) Mean duration of Oral-oral contacts for the morphine- (brown) and saline- (grey) treated mice, across LMT sessions. No difference between the two treatments was observed (2-way ANOVA: session: $F_{(4.137,78.61)}$=2.374, p=0.054; drug: $F_{(1,19)}$=0.003, p=0.958; drug x session: $F_{(4.137,78.61)}$=0.852, p=0.499). **c**) Mean number of rearing made in contact for the morphine- (brown) and saline- (grey) treated mice, across LMT sessions. No difference between the two treatments was observed (2-way ANOVA: session: $F_{(2.483, 47.17)}$=2.869, p=0.056; drug: $F_{(1,19)}$=0.8464, p=0.369; drug x session: $F_{(2.483, 47.17)}$=1.551, p=0.219). **d**) Mean number of rearing made isolated for the morphine- (brown) and saline- (grey) treated mice, across LMT sessions. Only a session effect between the two treatments was observed (2-way ANOVA: session: $F_{(3.208, 60.95)}$=7.612, p=0.0002; drug: $F_{(1,19)}$=0.009, p=0.924; drug x session: $F_{(3.208, 60.95)}$=2.445, p=0.069). **e**) Mean number of contacts made when side by side for the morphine- (brown) and saline- (grey) treated mice, across LMT sessions. No difference between the two treatments was observed (2-way ANOVA: session: $F_{(3.623, 68.84)}$=1.353, p=0.261; drug: $F_{(1,19)}$=1.581, p=0.224; drug x session: $F_{(3.623, 68.84)}$=2.051, p=0.103).). **f**) Mean number of breaks initiated after contact for the morphine- (brown) and saline- (grey) treated mice, across LMT sessions. Only a treatment effect between the two treatments was observed (2-way ANOVA: session: $F_{(3.466, 65.85)}$=1.982, p=0.117; drug: $F_{(1,19)}$=4.526, p=0.047; drug x session: $F_{(3.466, 65.85)}$=1.469, p=0.227). **g**) Mean time an individual follows the interactor for the morphine- (brown) and saline- (grey) treated mice, across LMT sessions. No difference between the two treatments was observed (2-way ANOVA: session: $F_{(3.348, 66.96)}$=2.645, p=0.050; drug: $F_{(1,19)}$=0.385, p=0.542; drug x session: $F_{(3.348, 66.96)}$=1.163, p=0.333). Data are represented as mean ± SEM, morphine n=11 and Saline control n=10.

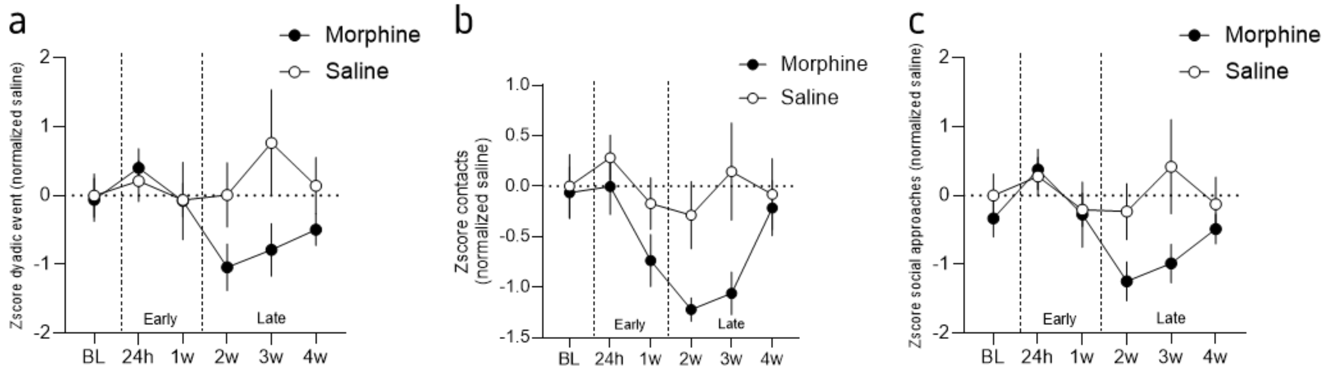

Supplementary Figure 3. a) Mean Z-score of the dyadic events, normalized from the saline-treated mice, across LMT sessions (2-way ANOVA: session: $F_{(3.333,63.33)}$=1.511, p=0.2167; drug: $F_{(1,19)}$=1.522, p=0.232; drug x session: $F_{(3.333,63.33)}$=2.484, p=0.063). **b**) Mean Z-score of the contacts, normalized from the saline-treated mice, across LMT sessions (2-way ANOVA: session: $F_{(3.972,75.47)}$=5.423, p=0.0007; drug: $F_{(1,19)}$=2.847, p=0.108; drug x session: $F_{(3.972,75.47)}$=2.642, p=0.041). **c**) Mean Z-score of the social approaches, normalized from the saline-treated mice, across LMT sessions (2-way ANOVA: session: $F_{(3.544,67.34)}$=3.280, p=0.0.02; drug: $F_{(1,19)}$=1.812, p=0.194; drug x session: $F_{(3.544,67.34)}$=2.355, p=0.07). Data are represented as mean ± SEM, morphine n=11 and Saline control n=10.

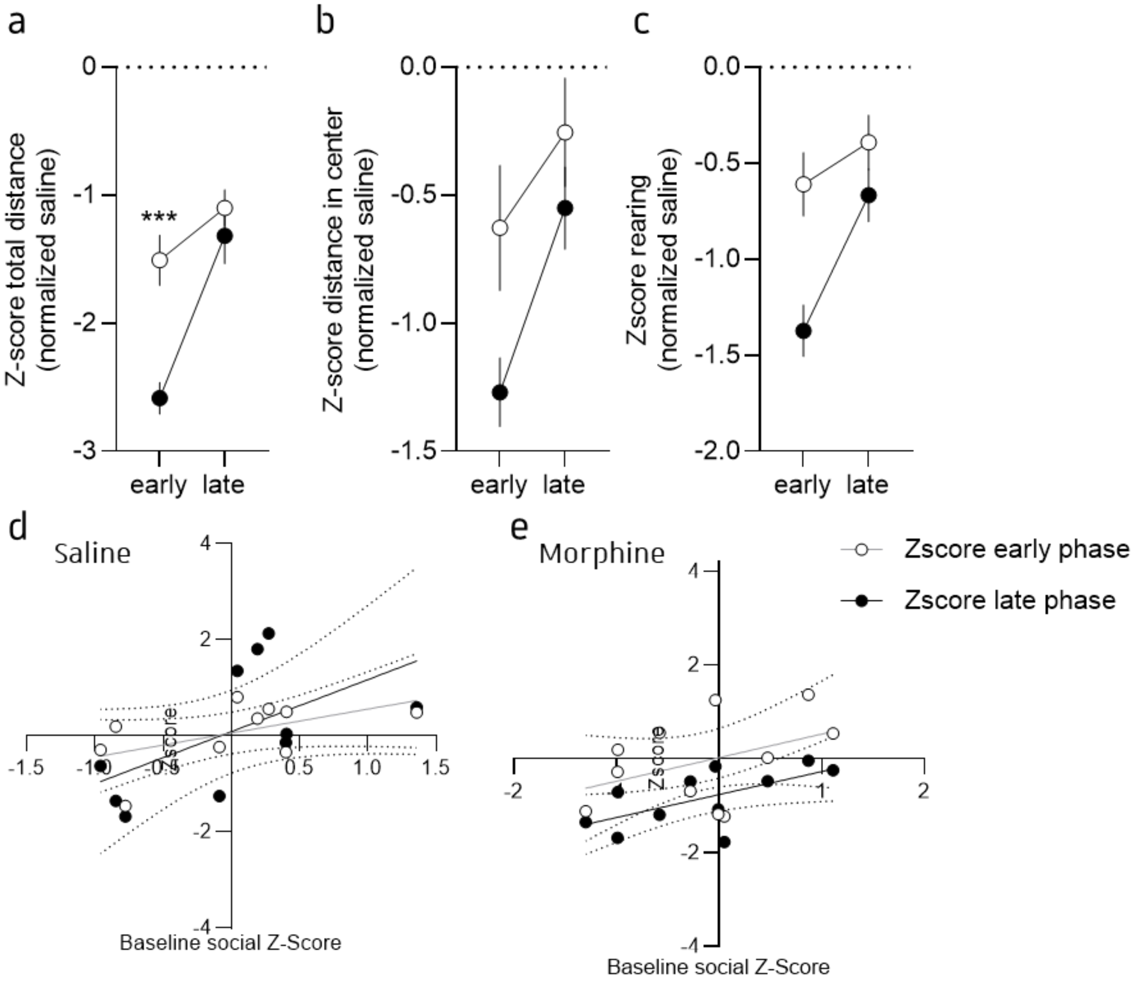

Supplementary Figure 4. a) Mean Z-score of the total distance travelled during habituation, normalized from the saline-treated mice, across LMT sessions (2-way ANOVA: session: $F_{(1,19)}$=41.96, p<0.0001 drug: $F_{(1,19)}$=9.422, p=0.0063; drug x session: $F_{(1,19)}$=11.06, p=0.004). **b**) Mean Z-score of the distance travelled in the center of the OF during habituation, normalized from the saline-treated mice, across LMT sessions (2-way ANOVA: session: $F_{(1,19)}$=17.65, p=0.0005 drug: $F_{(1,19)}$=4.030, p=0.059; drug x session: $F_{(1,19)}$=1.770, p=0.199). **c**) Mean Z-score of the number of rearing during habituation, normalized from the saline-treated mice, across LMT sessions (2-way ANOVA: session: $F_{(1,19)}$=28.64, p<0.0001 drug: $F_{(1,19)}$=7.833, p=0.012; drug x session: $F_{(1,19)}$=7.983, p=0.011). **d**) Linear regression showing the absence of correlation between the baseline social Z-score and the early and late social Z-score for saline-treated mice (early: r2=0.284, p=0.113; white circles, late: r2=0.321, p=0.88; brown circles). **e**) Linear regression showing a positive correlation between baseline social Z-score and the social Z-score at late (r2=0.384, p=0.042; brown circles) but not early stage (r2=0.174, p=0.202; white circles) for morphine-treated mice. Data are represented as mean ± SEM, morphine n=11 and Saline control n=10.
